# Sugammadex vs Neostigmine, a Comparison in Reversing Neuromuscular Blockade: A Narrative Review

**DOI:** 10.7759/cureus.65656

**Published:** 2024-07-29

**Authors:** Shafaque Maqusood, Amol Bele, Neeta Verma, Sambit Dash, Dushyant Bawiskar

**Affiliations:** 1 Anaesthesiology, Jawaharlal Nehru Medical College, Datta Meghe Institute of Higher Education and Research, Wardha, IND; 2 Sports Medicine, Abhinav Bindra Targeting Performance, Bangalore, IND

**Keywords:** train-of-four ratios, anaesthesia, neuromuscular blocking agents, neostigmine, sugammadex

## Abstract

The use of neuromuscular blocking agents (NMBA) has grown due to the development of laparoscopic and minimally invasive procedures. Respiratory insufficiency, an elevated risk of aspiration, postoperative pulmonary complications, and subsequent reintubation are among the risks linked to the residual block. The normal clinical practice calls for the pharmacologic "reversal" of these agents with either sugammadex or neostigmine prior to extubation. The administration of neostigmine is linked to a number of potential complications. In response, anaesthesiologists have begun to prescribe sugammadex more frequently for treating residual block and reversing blockade with NMBA. This review article compares and assesses neostigmine and sugammadex thoroughly in order to determine the extent to which they work as agents to reverse neuromuscular blockade. The review's findings highlight sugammadex's considerable advantages - Sugammadex's ability to quickly and reliably achieve desired train-of-four (TOF) ratios - over neostigmine in reversing neuromuscular blockade in a variety of surgical settings. In contrast, neostigmine's limitations regarding efficacy and rate of reversal were consistently noted in all of the reviewed studies, despite the fact that it is still widely used due to its lower cost and extensive clinical experience. Sugammadex is a superior option for reversing neuromuscular blockade, but incorporating it into standard clinical practice necessitates carefully weighing its potential benefits and drawbacks. Sugammadex provides notable benefits over neostigmine in terms of speed, predictability, and safety.

## Introduction and background

Across the world, the emergency department, operating room, and intensive care unit settings frequently employ a class of drugs known as neuromuscular blocking agents (NMBAs). NMBAs can be used for a variety of purposes, including routine use during surgical procedures, acute respiratory distress syndrome (ARDS), entire-body paralysis for specific medical indications, and rapid sequence intubation for respiratory failure [1]. Respiratory insufficiency, an elevated risk of aspiration, postoperative pulmonary complications, and subsequent reintubation are among the risks linked to the residual block [2]. The majority of adult patients receiving endotracheal intubation under general anaesthesia are administered an NMBA, such as vecuronium or rocuronium. The use of deep NMBA has grown due to the development of laparoscopic and minimally invasive procedures [3,4]. Deep NMBAs are a subset of NMBAs that produce profound muscle relaxation, resulting in more extensive paralysis compared to NMBAs. The normal clinical practice calls for the pharmacologic "reversal" of these agents with either sugammadex or neostigmine prior to extubation. Despite this, due to patient-to-patient variability in pharmacologic response and provider variation in care, over 60% of patients still exhibit objective evidence of residual NMB [5]. Following non-cardiac inpatient surgery, major postoperative pulmonary complications are common that are expensive and fatal. Major pulmonary complications affect about 5% of patients and are associated with a higher mortality rate and additional costs of $100,000 per instance [6]. Residual NMB following surgery is still a common modifiable risk factor for significant postoperative pulmonary complications despite advancements in surgical technique, perioperative procedures, and patient selection [7].

Neostigmine combined with an anticholinergic agent was the preferred reversal agent for the residual block before the approval of sugammadex in late 2015. The administration of neostigmine is linked to a number of potential complications. In response, anaesthesiologists have begun to prescribe sugammadex more frequently for treating residual block and reversing blockade with NMBAs [8]. Neostigmine is the most widely used medication in this class of drugs and is also the most potent anticholinesterase inhibitor. The maximum amount of neostigmine and pyridostigmine that is advised for NMBA reversal is 70 mcg/kg and 350 mcg/kg, respectively. Through the inhibition of acetylcholinesterase and butyrylcholinesterase enzymes, anticholinesterase inhibitors cause NMBA reversal. The amount of acetylcholine at the receptor junction generally rises as a result of the inhibition of these molecules. Eventually, this leads to the restoration of regular motor nerve impulses and the ability of muscles to function [9].

Anticholinesterase inhibitors accelerate the process by which NMBAs cease to have paralysing effects, but they are linked to a number of potentially detrimental side effects. The negative consequences stemming from the activation of muscarinic receptors comprise bradycardia, hypotension, dysrhythmias, bronchoconstriction, salivary gland stimulation, muscle weakness, and postoperative nausea and vomiting. To mitigate these effects, it is necessary to co-administer an anticholinergic agent such as glycopyrrolate, edrophonium, or atropine with anticholinesterase inhibitors [10]. The substance sugammadex is a modified γ-cyclodextrin that functions by binding to amino steroidal non-depolarising NMBAs. It, being the first selective relaxant binding agent, represents a novel class of therapeutic agents [11]. The way sugammadex works is primarily due to its capacity to encapsulate free amino steroidal NMBA molecules, which prevents the molecules from binding to and blocking acetylcholine receptors. As a result of these encapsulated substances diffusing away from acetylcholine receptors and reducing the concentration of free NMBA molecules there, bound NMBA molecules dissociate from acetylcholine receptors [12,13].

Sugammadex only affects amino steroidal compounds; benzylisoquinoline molecules are unaffected by it. When sugammadex is compared to the anticholinesterase inhibitor medication class, there is an improved reversal of residual block, even for patients with profound degrees of paralysis, due to its binding affinity [14,15]. Sugammadex dosage recommendations are contingent on both the degree of neuromuscular blockade and the NMBA that requires reversal. The advised dosages of sugammadex correspond to what is needed to hasten a patient's recovery from a neuromuscular block - 0.9 TOF ratio on average after three minutes [16]. This review article compares and assesses neostigmine and sugammadex thoroughly in order to determine the extent to which they work as agents to reverse neuromuscular blockade. The study intends to evaluate the effectiveness, safety profiles and mechanistic insights of the current research findings by synthesising them.

## Review

Methodology

Search Strategies

A comprehensive search strategy was deployed in the literature review, involving multiple databases such as PubMed, Scopus, Web of Science and Google Scholar. This involved “sugammadex,” “neostigmine,” “neuromuscular blocking agents,” “anaesthesia,” and “train-of-four ratios” as the keywords used. To combine search terms and refine search results Boolean operators (AND, OR) were used. Only articles published in English from January 2010 to October 2023 were included in this search to capture recent and relevant studies.

Criteria for Inclusion and Exclusion

Inclusion criteria were set up to identify studies that specifically involved the use of sugammadex and neostigmine, had quantitative data and patient outcomes, and were peer-reviewed. The exclusion criteria included studies about other drugs, case reports, review articles, editorials and studies without full text.

Extraction of Data and Its Synthesis

A total of 192 articles were initially identified using the search strategy. After removing duplicates, there were 145 articles. These papers were then screened based on titles and abstracts, giving a total of 56 papers for full-text review. Among these many papers that underwent thorough assessment against several inclusion/exclusion criteria, only 15 meet this standard; hence, they have been considered for detailed analysis. The data extracted from these studies consisted of study design, sample size, patient demographics, ventilation parameters, intraoperative and postoperative outcomes, as well as major findings contained in the articles themselves. Table 1 shows information about the articles reviewed for the study.

**Table 1:** Description of studies included in the review TOF: Train-of-four, NMB: Neuromuscular blockage, PONV: Post-operative nausea vomiting, PACU: Post-anaesthesia care unit, MO: Morbidly obese, PORC: Post-operative respiratory complication, ECG: Electrocardiogram, IOP: Intra-ocular pressure, TOFR: Train-of-four ratio, TFIO: Thickening fraction of internal oblique abdominal muscle, EMG: Electromyography, CBW: Corrected body weight, CVD: Cardiovascular disease

Sr. no	Author and year	Participants	Type of surgery	Intervention	Outcome measures	Conclusion
1	Blobner et al. (2010) [17]	98 adult patients	Any elective surgery	For tracheal intubation and neuromuscular blockade maintenance, patients were allocated at random to receive either rocuronium or vecuronium. For neuromuscular blockade reversal, they were given either sugammadex 2.0 mg kg(-1) or neostigmine 50 microgram kg (-1) (with glycopyrrolate 10 microgram kg(-1)).	Neuromuscular monitoring was performed using evoked acceleromyography of the adductor pollicis muscle.	The recovery time of the TOF ratio of 0.9 following sugammadex was notably shorter than that of neostigmine, at 1.5 minutes as compared to 18.6 minutes. Sugammadex patients recovered to a TOF ratio of 0.9 in 5 minutes, compared to 11% of neostigmine patients; this indicates that sugammadex patients had a more predictable response than neostigmine patients.
2	Adamus et al. (2011) [18]	22 adult patients undergoing	Extreme lateral interbody fusion (spine surgery)	Patients undergoing surgery for rocuronium block reversal were randomised to receive either sugammadex (2 mg/kg) or neostigmine (0.04 mg/kg).	Both the electromyographic response of the adductor pollicis muscle and TOF stimulation of the ulnar nerve at 15-s intervals were used.	In order to accurately identify lumbar nerve roots during surgery, the NMB needs to be reversed to a TOF ratio of at least 0.70 when using a stimulating current of 10 mA. Regarding a current at a 5 mA intensity, the corresponding TOF ratio ought to be 0.90. It is possible to reach these target TOF ratios with 0.04 mg/kg of neostigmine and 2 mg/kg of sugammadex.
3	Kheterpal et al. (2021) [19]	45,172 patients	Elective inpatient noncardiac surgical procedures with general anesthesia and endotracheal intubation receiving a nondepolarizing neuromuscular blockade agent	22,856 patients receiving sugammadex and 22,856 patients receiving neostigmine were matched and divided into 2 groups, they received 3.5% sugammadex and 4.8% neostigmine.	According to international consensus guidelines, the main outcome was a composite of postoperative pulmonary complications that were likely related to residual NMB. Pneumonia, respiratory failure, or other significant pulmonary complications are the possible outcomes.	Sugammadex use was linked to a statistically and clinically significant reduction in the incidence of major pulmonary complications in a generalizable cohort of adult patients undergoing inpatient surgery at US hospitals. While sugammadex quickly and efficiently restores neuromuscular tone without causing systemic anticholinergic activity, neostigmine is still the standard of care in most countries due to decades of experience and sugammadex's higher cost.
4	Brueckmann et al. (2015) [20]	154 adult patients	Abdominal surgery	Subjects receiving general anaesthesia with rocuronium-induced neuromuscular blockade during elective laparoscopic or open abdominal surgery, sugammadex (2 or 4 mg kg (-1)) or neostigmine/glycopyrrolate (dosing per usual care practice) were given to patients at random.	During surgery, the degree of neuromuscular blockade was assessed by accelerator-myography-based neuromuscular monitoring at the adductor pollicis muscle.	The duration between the administration of reversal agents and the readiness for operating room discharge was shortened in the case of sugammadex compared to standard care, suggesting a quicker recovery of neuromuscular function in the operating room and a higher level of surgical efficiency. It is more economical to reverse neuromuscular blockade completely if muscles heal quickly. Strength can be used in routine clinical practice to shorten recovery times. Top of Form
5	Carron et al. (2013) [6]	40 female patients who are MO.	Morbidly obese patients undergoing elective surgery.	NMB was obtained by using rocuronium. Sugammadex or neostigmine plus atropine was used to achieve total reversal of NMB at the conclusion of the surgical procedure. Sugammadex (2 mg/kg) or neostigmine (0.04 mg/kg) was given.	At the time of PACU admission, an accelerator-myographic low stimulation current (30 mA) was used to assess TOFR in order to determine the extent of residual NMB.	Sugammadex reduces the chance of PORC, speeds up the recovery process from profound NMB, and enables MO patients to resume their mobility more quickly. Alongside fast-acting, short-acting volatile anaesthetics and sugammadex and opioids enable bariatric fast-track surgery.
6	Castro et al. (2014) [21]	88 MO patients	Bariatric surgery	Patients were split into two groups in order to examine the impact of sugammadex 2 mg/kg on postoperative pain: those who received or neostigmine 0.05 mg/kg.	A visual analogue scale was employed to measure pain. Four separate times were considered for evaluation: when the patient first arrived in the PACU, 30 minutes later, 60 minutes later, and right before returning to the surgery ward.	Sugammadex is linked to a reduction in pain experienced in the PACU. Sugammadex's "opioid-sparing" action, along with a decrease in PONV and a quicker discharge from the PACU, make it an essential medication for patients of this kind and permits expedited surgery in the MO.
7	Cheong et al. (2015) [22]	120 patients	Any elective surgery.	One of four groups (Groups S2, S1, SN, and N) was randomly assigned to receive intravenous sugammadex 2 mg/kg. Group S1 received sugammadex 1 mg/kg, Group SN received sugammadex 1 mg/kg plus neostigmine 50 µg/kg + glycopyrrolate 10 µg/kg, and Group N received neostigmine 50 µg/kg + glycopyrrolate 10 µg/kg for the purpose of reversing the neuromuscular blockade.	When the patients entered the operating room, the accelerometer was fixed on the ipsilateral thumb, two stimulating electrodes of the TOF-Watch® were placed over the ulnar nerve on the wrist at intervals of 3 to 4 cm, and the ECG, blood pressure, and pulse oximetry were recorded.	Neostigmine and sugammadex together may help to shorten the recovery period and lower the dosage of sugammadex required for the reversal of rocuronium-induced moderate neuromuscular blockade. However, when using sugammadex in conjunction with neostigmine, the clinical anesthesiologist needs to take into account the higher risk of systemic muscarinic side effects.
8	Flockton et al. (2008) [23]	84 adult patients	Any elective surgery.	The adult surgical patients were randomly assigned to receive sugammadex 2.0 mg kg21 for rocuronium-induced block reversal or neostigmine 50 mg kg21 for cisatracurium-induced block reversal (0.15 mg kg21).	Acceleromyography was used to track neuromuscular function (TOF-Watch SX).	In surgical patients, the rocuronium-induced neuromuscular block can be quickly and successfully reversed with sugammadex 2.0 mg kg21 given at the reappearance of T2. When it came to reversing rocuronium-induced neuromuscular block, sugammadex was noticeably quicker than neostigmine when it came to reversing cisatracurium-induced block. Neostigmine and sugammadex were both well tolerated and safe.
9	Gaszynski et al. (2012) [24]	70 MO patients	MO patients undergoing surgery requiring general anaesthesia.	Patients who required general anaesthesia and were given rocuronium for muscle relaxation were divided into two groups at random. The first group received sugammadex 2 mg kg21 at the end of the anaesthetic procedure, while another group received neostigmine 0.05 mg kg21 of CBW.	After surgery, the patient's response was measured by a TOF score.	The study concludes with the finding that sugammadex 2.0 mg kg21 CBW given at the onset of T2 can effectively and quickly reverse rocuronium-induced neuromuscular block and prevent PORC in patients who are morbidly obese. Compared to neostigmine, sugammadex reversed rocuronium-induced neuromuscular block much more rapidly. Sugammadex was well-tolerated and safe. Neostigmine did not completely prevent PORC from occurring, but it did cause some side effects.
10	Geldner et al. (2012) [25]	140 adult patients	Any laparoscopic surgery	Patients were randomised to receive atropine (10 mg.kg^-1^) plus either neostigmine (50 µg. kg^-1^) or sugammadex (4 mg.kg^-1^).	Acceleromyography was used for continuous neuromuscular monitoring at the adductor pollicis muscle following the induction of anaesthesia.	Sugammadex neuromuscular blockade reversal at a post-tetanic count of 1-2 after rocuronium was well tolerated and led to a faster recovery of the TOF ratio to 0.9 compared with neostigmine administered at the reappearance of T2 in patients undergoing laparoscopic surgery under propofol anaesthesia. Consequently, sugammadex may enable quick and painless reversal of deep neuromuscular blockade following surgery.
11	Hakimoglu et al. (2016) [26]	60 adult patients undergoing	Arthroscopic surgery	Two groups were randomly assigned to the patients. Following the procedure, Group 1 received neostigmine (50 mg/kg) plus atropine (15 mg/kg), while Group 2 received sugammadex (4 mg/kg) to reverse the neuromuscular block.	Throughout the procedure, standard monitoring was carried out, including an ECG, noninvasive blood pressure readings, heart rate, and peripheral arterial oxygen saturation. Train-of-four (TOF-Watch SX; Organon Ireland Ltd., Dublin, Ireland) was used to monitor the degree of neuromuscular blockade in addition to routine monitoring.	The sugammadex group's post-extubation IOP values resembled those of the neostigmine atropine group. Furthermore, our study found that the sugammadex group experienced a shorter extubation time than the neostigmine atropine group, which is consistent with earlier research.
12	Kaufhold et al. (2016) [27]	99 adult patients	Any elective surgery	Ninety-nine patients received either saline or sugammadex at doses of 0.25, 0.5, 0.75, 1.0, and 1.25 mg kg^−^1, neostigmine at doses of 10, 25, 40, 55, and 70 µg kg^−1^ in a mixture containing 1 µg glycopyrrolate for every 5 µg neostigmine.	Using the NMT module in an S/5 GE Datex Light monitor, evoked EMG of the adductor pollicis muscle was used for neuromuscular monitoring in accordance with international consensus guidelines.	Sugammadex (∼0.26 mg kg−1) can reverse a TOFR from 0.2 to ≥0.9 in 10 minutes in 95% of patients, but neostigmine was ineffective in this regard. Sugammadex (∼0.50 mg kg−1) can also reverse a residual neuromuscular block at a TOFR≥0.2 in 95% of patients quickly (within 5 min).
13	Kizilay et al. (2016) [28]	99 patients with class 2 or 3 CVD.	Non-cardiac surgery	Following surgery, patients in group 1 were given IV sugammadex at a dose of 3 mg/kg when the T2 level in the train of four resurfaced. After the procedure, patients in the second group received IV 0.03 mg/kg neostigmine when their T2 level returned and was tracked by a nerve-muscle stimulator.	A nerve muscle stimulator was used.	The study compared the hemodynamic effects of sugammadex and neostigmine in patients with heart conditions who had non-cardiac surgery. Between the two groups, they could not find any differences in the QT interval. Hemodynamic parameters increased significantly in both groups, but the increase was more pronounced in neostigmine-treated patients. Sugammadex is a potentially safe option to reverse neuromuscular blockade in patients with heart conditions undergoing non-cardiac surgery.
14	Choi et al. (2017) [29]	44 adult patients undergoing laryngeal microsurgery	Elective laryngeal micro-surgery	Divided into two groups at random: the moderate block group, which included rocuronium 0.45 mg kg^-1^ with neostigmine (50 µg.kg^~1^ with glycopyrrolate 10 µg.kg^-1^) reversal, and the deep block group, which included rocuronium 0.90 mg kg^~1^ with sugammadex (4 mg.kg^~1^) reversal.	The tracking programme TOF-Watch SX was utilised to gather train-off data automatically.	Patients receiving deep neuromuscular blockade with sugammadex as the reversal medication during elective laryngeal microsurgery, have significantly better surgical outcomes and a shorter recovery period when compared to rocuronium dosage reduction with neostigmine reversal.
15	Huang et al. (2023) [30]	58 adult patients	Micro-laryngeal surgery	Following surgery, patients were given either sugammadex (2 mg·kg^−1^) or neostigmine (50 μg·kg^−1^, maximum 5 mg) in combination with atropine (25 μg·kg^−1^, maximum 2.5 mg).	Using a TOFR recovery to 0.9, the TFIO and diaphragm excursion, which represent expiratory and inspiratory muscle strength, were measured by ultrasonography three times before induction (baseline), and 30 minutes after the PACU arrived. The shift in TFIO from baseline to TOFR ≥0.9 was the main result.	Immediately following extubation, sugammadex improves the recovery of expiratory muscle strength more thoroughly than neostigmine. It is necessary to provide additional evidence of the connection between the treatment allocation and the recovery of expiratory muscle strength after extubation that lasts longer than 30 minutes.

A study by Blobner et al. involved 98 adult patients who underwent tracheal intubation and neuromuscular blockade maintenance using either rocuronium or vecuronium. For neuromuscular blockade reversal, patients were given either sugammadex or a neostigmine-glycopyrrolate mixture. Neuromuscular monitoring was performed using evoked acceleromyography of the adductor pollicis muscle. The study found that the recovery time to a TOF (train-of-four) ratio of 0.9 following sugammadex administration was significantly shorter than that following neostigmine, with times of 1.5 minutes versus 18.6 minutes, respectively. Additionally, 95% of patients treated with sugammadex recovered to a TOF ratio of 0.9 within 5 minutes, compared to only 11% of those treated with neostigmine, indicating a more predictable and rapid response with sugammadex [17]. Adamus et al. (2011) did a study that included 22 adult patients undergoing extreme lateral interbody fusion for spine surgery. Patients were randomized to receive either sugammadex or neostigmine for rocuronium block reversal. To accurately identify lumbar nerve roots during surgery, it was necessary to reverse the neuromuscular block to a TOF ratio of at least 0.70 with a stimulating current of 10 mA and 0.90 with a current of 5 mA. These target TOF ratios could be achieved with 0.04 mg/kg of neostigmine and 2 mg/kg of sugammadex [18].

In a study by Kheterpal et al., 45,172 patients were matched, with 22,856 patients receiving sugammadex and an equal number receiving neostigmine. The study compared the incidence of postoperative pulmonary complications likely related to residual neuromuscular blockade. The main outcomes included pneumonia, respiratory failure, or other significant pulmonary complications. The use of sugammadex was associated with a significant reduction in major pulmonary complications, demonstrating its effectiveness in quickly and efficiently restoring neuromuscular tone without causing systemic anticholinergic activity, which is a concern with neostigmine [19]. A study by Brueckmann et al. involved 154 adult patients undergoing elective laparoscopic or open abdominal surgery under general anaesthesia with rocuronium-induced neuromuscular blockade. Patients were randomized to receive either sugammadex or a neostigmine/glycopyrrolate mixture for neuromuscular blockade reversal. Neuromuscular monitoring was performed using acceleromyography of the adductor pollicis muscle. The time between the administration of reversal agents and readiness for discharge from the operating room was significantly shorter with sugammadex, indicating quicker recovery of neuromuscular function and improved surgical efficiency [20].

In the study by Carron et al., 40 morbidly obese female patients undergoing surgery with rocuronium-induced neuromuscular blockade were randomized to receive either sugammadex or neostigmine plus atropine for total reversal of neuromuscular blockade at the end of the surgical procedure. Upon admission to the PACU (post-anesthesia care unit), neuromuscular function was assessed using acceleromyography with low stimulation current (30 mA) to determine the extent of residual neuromuscular blockade. The study found that sugammadex reduced the chance of postoperative residual curarization (PORC), sped up recovery from profound neuromuscular blockade, and enabled quicker mobility, facilitating fast-track bariatric surgery [6]. The study by Castro et al. involved 88 morbidly obese patients undergoing bariatric surgery who were randomized to receive either sugammadex or neostigmine for neuromuscular blockade reversal. Pain levels were assessed using a visual analogue scale at four-time points indicating that at four time periods, the assessment of pain took place: upon arrival in the PACU, 30 minutes later, 60 minutes later, and before returning to the surgical ward. The study found that sugammadex was associated with reduced postoperative pain, likely due to its "opioid-sparing" effect. It also contributed to a decrease in postoperative nausea and vomiting and quicker discharge from the PACU [21].

The studies collectively demonstrate that sugammadex consistently outperforms neostigmine in reversing neuromuscular blockade across a variety of patient populations. Sugammadex offers faster recovery times to a TOF ratio of 0.9, indicative of adequate neuromuscular function recovery, and reduces the incidence of PORC and associated pulmonary complications. For example, Flockton et al. (2008) and Gaszynski et al. (2012) highlight that sugammadex can quickly and effectively reverse rocuronium-induced neuromuscular block, showing significantly faster performance than neostigmine [23,24]. In addition to faster recovery times, sugammadex demonstrates other advantages, such as reduced postoperative pain and nausea, as noted by Castro et al., and more stable hemodynamic responses in cardiovascular patients, as reported by Kizilay et al. These benefits are especially pronounced in high-risk populations, such as the morbidly obese and those with cardiovascular disease. Studies like those by Cheong et al. and Hakimoglu et al. also illustrate that sugammadex can effectively and safely reverse neuromuscular block with fewer side effects and shorter extubation times compared to neostigmine [28,29].

The ability of sugammadex to reverse deeper levels of neuromuscular blockade further underscores its superiority in various clinical scenarios. Studies such as those by Geldner et al. and Kaufhold et al. reveal that sugammadex can quickly achieve a TOF ratio of 0.9, even from deeper levels of blockade, while neostigmine struggles with this task. These findings collectively affirm that sugammadex not only enhances the efficiency of surgical procedures but also improves patient safety and comfort, representing a significant advancement in anaesthesia management [30].

Discussion

NMBAs facilitate procedures like endotracheal intubation and mechanical ventilation. They also help surgeons achieve a superior surgical field of view. Postoperative residual neuromuscular blockade, however, may raise the risk of respiratory complications and postoperative pulmonary diseases, including atelectasis, low oxygen saturation, and upper airway obstruction. These could result in unexpected reintubation, longer hospital stays and, in extreme cases, potentially fatal complications [31,32]. Before sugammadex was accepted in 2015, anticholinesterase medications were the only way for clinicians to reverse residual neuromuscular blockade.

Neostigmine administration has been linked to various side effects that necessitate the coadministration of anticholinergic medication, and it has not been associated with a rapid or predictable reversal of residual blocking. On the other hand, sugammadex consistently reverses neuromuscular blockade and restores muscle function. However, it also has some disadvantages, such as it can cause bradycardia and cardiovascular effects, hypersensitivity reactions, and it has high costs [33]. The review's findings highlight sugammadex's considerable advantages over neostigmine in reversing neuromuscular blockade in a variety of surgical settings. Sugammadex's ability to quickly and reliably achieve desired TOF ratios is especially noteworthy, as evidenced by studies like Blobner et al. and Adamus et al. [17,18]. Compared to neostigmine, sugammadex consistently provides faster recovery times and more consistent reversal outcomes, which is consistent with the objectives of improved patient safety and effective surgical management. Critical factors to take into account are the wider clinical advantages of sugammadex, which include its ability to lessen postoperative pulmonary complications linked to residual neuromuscular blockade.

These results enhance patient recovery while also helping to lower medical expenses related to prolonged hospital stays. But even with these obvious benefits, there are still obstacles to sugammadex adoption. The most important of these are financial since it is more expensive to acquire than neostigmine. Numerous studies have observed that institutional preferences originating from decades of neostigmine use also pose obstacles to the widespread adoption of sugammadex.

## Conclusions

Sugammadex is a good option for reversing neuromuscular blockade, but incorporating it into standard clinical practice necessitates carefully weighing its potential benefits and drawbacks. Sugammadex is thought to provide notable benefits over neostigmine in terms of speed, predictability, and safety, according to the evidence reviewed. To further support sugammadex's ideal use in perioperative care, future research should continue to examine the cost-effectiveness and long-term clinical outcomes related to its use. When choosing neuromuscular blockade reversal agents, clinicians and other healthcare professionals are advised to carefully consider these variables in order to maximise patient outcomes while negotiating institutional limitations and financial considerations.
